# Report of the Medical Research Committee

**Published:** 1916-12-02

**Authors:** 


### REPORT OF THE MEDICAL RESEARCH COMMITTEE.

The Medical Research Committee, like many other organisations, has been largely drawn from its peaceful orbit by the disturbing influence of the war, and its latest report affords convincing evidence of this influence. The Committee, it will be remembered, took origin as part of the scheme of National Insurance, and it was intended to explore all the problems of preventative and curative medicine which experience of this scheme should propose. In adopting and endorsing the Committee the State gave direct support to a plan of medical investigation on rigorously scientific lines; and whatever may be said of other parts of the Insurance scheme, this one certainly commanded general sympathy. Nor was there any reason to doubt that the expenditure would justify itself, seeing that insurance liabilities take a very practical shape, and that the extent of these would be sensibly modified by improved efficiency in therapeutics and prophylaxis. The prospect of influential and enduring work was thus before the Committee, and much was expected at its hands. Though genius is not to be bred by such means, industry, system, organisation, and co-ordination can certainly be commanded; and, had all gone well, many interesting problems would doubtless have been defined, and some perhaps would have been brought to solution.

The calm and peaceful programme of the Committee has been destroyed, at least for the time being, by the rude note of war. It is not the medical problems of industrial life which are first in urgency when millions of men stand in battle array and struggle for mastery. The claim is for help under new conditions and in different circumstances. Consequently, the Committee has become in substance, if not in name, part of the national military organisation; and the claims on its resources have been abundant, for never before has it been so clearly realised that medical knowledge and co-operation are a large factor in the efficiency of armies in the field. Not only is this true, but it has to be admitted that the war has raised new questions, and has thrown doubt on some issues which previously had been regarded as settled beyond dispute. The Committee was in existence as an organised machine for dealing with difficult and uncertain medical problems, and, very properly, when the crisis came its energies were commandeered for Army purposes. To say that it has been helpful in these circumstances is a mild term to employ. Indeed, it has proved itself an essential part of our military medical machinery, and had it not existed someone would have had to invent it. In some quarters there is a movement to make more intimate the relations between the Committee and the Army authorities. If this will promote more easy working we should recognise that the circumstances of the hour justify it. But, speaking generally, we should be inclined to say that the less an organisation of this order is bound by official ties the better. In the very nature of things, there must be some formal control of a Committee which draws its financial ability from Government sources. But enterprise, initiative, and ingenuity depend in the last analysis on personal freedom, and it is these qualities which are especially necessary for the successful penetration of scientific questions. Everything must yield to the urgency of the present moment, but our own disposition is to hope that the Research Committee will be able to continue a career of independence and of freedom. That it has done much to help the Army the present report shows, and we doubt whether the work will be improved by the official control of the War Office. In any event, it is to the benefit of the nation, as well as of science, that medical investigation should be skilfully organised to practical ends, and this, in principle, is the scheme on which the Committee is engaged. With regard to the actual work accomplished, the details are too numerous and too technical to be summarised here. The field is a very wide one. It ranges from the treatment of military wounds to the selection of diet for munition workers, from the disinfection of hospital ships to the hygienic relations of milk. We will make no attempt to appraise the value of all these records and statistics. But this may certainly be kept in mind, that the aim of the whole enterprise is to secure practical results and not mere academic propositions. The intention is to render medicine in a larger and larger degree a body of knowledge capable of affording help and succour to suffering humanity.

